# A Prospective Study of Organochlorines in Adipose Tissue and Risk of Non-Hodgkin Lymphoma

**DOI:** 10.1289/ehp.1103573

**Published:** 2011-08-26

**Authors:** Elvira Vaclavik Bräuner, Mette Sørensen, Eric Gaudreau, Alain LeBlanc, Kirsten Thorup Eriksen, Anne Tjønneland, Kim Overvad, Ole Raaschou-Nielsen

**Affiliations:** 1 Institute of Cancer Epidemiology, Danish Cancer Society, Copenhagen, Denmark; 2 Centre de toxicologie du Québec, Institut National de Santé Publique du Québec, Quebec City, Quebec, Canada; 3 Department of Epidemiology, School of Public Health, Aarhus University, Aarhus, Denmark

**Keywords:** adipose tissue, case–cohort, non-Hodgkin lymphoma, organochlorines

## Abstract

Background: Exposure to organochlorines has been examined as a potential risk factor for non-Hodgkin lymphoma (NHL), with inconsistent results that may be related to limited statistical power or to imprecise exposure measurements.

Objective: Our purpose was to examine associations between organochlorine concentrations in prediagnostic adipose tissue samples and the risk of NHL.

Methods: We conducted a case–cohort study using a prospective Danish cohort of 57,053 persons enrolled between 1993 and 1997. Within the cohort we identified 256 persons diagnosed with NHL in the population-based nationwide Danish Cancer Registry and randomly selected 256 subcohort persons. We measured concentrations of 8 pesticides and 10 polychlorinated biphenyl (PCB) congeners in adipose tissue collected upon enrollment. Associations between the 18 organochlorines and NHL were analyzed in Cox regression models, adjusting for body mass index.

Results: Incidence rate ratios and confidence intervals (CIs) for interquartile range increases in concentrations of dichlorodiphenyltrichlorethane (DDT), *cis*-nonachlor, and oxychlordane were 1.35 (95% CI: 1.10, 1.66), 1.13 (95% CI: 0.94, 1.36), and 1.11 (95% CI: 0.89, 1.38), respectively, with monotonic dose–response trends for DDT and *cis*-nonachlor based on categorical models. The relative risk estimates were higher for men than for women. In contrast, no clear association was found between NHL and PCBs.

Conclusion: We found a higher risk of NHL in association with higher adipose tissue levels of DDT, *cis*-nonachlor, and oxychlordane, but no association with PCBs. This is the first study of organochlorines and NHL using prediagnostic adipose tissue samples in the exposure assessment and provides new environmental health evidence that these organochlorines contribute to NHL risk.

Non-Hodgkin lymphomas (NHLs) are a heterogeneous group of B- and T-cell neoplasms arising primarily in the lymph nodes. Incidence rates have nearly doubled in Western countries since the 1970s, although no further rise has been observed since the mid-1990s (Dreiher and Kordysh 2006). Established risk factors include genetic susceptibility and previous history of malignant disease (Wang et al. 2007). Immunological conditions are also related to this group of cancers, as evidenced by a more than 100-fold increased risk of NHL among organ transplant recipients receiving immunosuppressive therapy (Fisher and Fisher 2004) and an increased incidence among subjects with primary immunodeficiency and autoimmune diseases (Vineis et al. 2000). However, these factors, in combination with longer life spans and changes in diagnostic patterns, cannot explain all cases, and many researchers speculate that the increase is partly due to exposure to environmental agents, including organochlorines (Hardell and Axelson 1998).

Dichlorodiphenyltrichlorethane (DDT) and other organochlorines are carcinogenic in animals (International Agency for Research on Cancer 1987), and the production and environmental release of these compounds with subsequent bioaccumulation accelerated during the decades after 1945 (World Resources Institute 1990), coinciding with increasing incidence rates for NHL in Western populations. Organochlorines are ubiquitously present as a mixture of mother compounds and metabolites in the environment and are characterized by high lipid solubility, environmental persistence, bioaccumulation (Agency for Toxic Substances and Disease Registry 1993), and long biological half-lives in human tissue (Wolff et al. 2000). Several studies have described immunotoxicity of selected organochlorines (Heilmann et al. 2006; Reed et al. 2004; Tryphonas et al. 2003).

The evidence relating organochlorines to NHL risk has been reviewed (Dreiher and Kordysh 2006) and the association remains unclear, with some studies reporting associations and others finding none. Reasons for these discrepancies may include low statistical power or imprecise exposure measurements, such as surrogate measures including occupation or self-reported questionnaires gathered after diagnosis. More recent studies have evaluated biological exposure measurements and have more consistently reported associations between NHL risk and various organochlorine pesticides and polychlorinated biphenyl (PCB) congeners (Bertrand et al. 2010; Cantor et al. 2003; De Roos et al. 2005; Engel et al. 2007; Laden et al. 2010; Quintana et al. 2004; Spinelli et al. 2007). However, the specific compounds identified were not consistent among the studies, and different biological media were used. Questions pertaining to the best quantification of biological exposure have been raised (Aronson et al. 2000; Whitcomb et al. 2005), and adipose tissue samples have been regarded by various authors (Allam and Lucena 2001; Hardell et al. 1996; Quintana et al. 2004) as the preferred indicator of human exposure because organochlorine levels are known to be higher in adipose tissue and more representative of the cumulative internal exposure. However, only two studies have previously measured organochlorines in adipose tissue (Hardell et al. 1996; Quintana et al. 2004), and in both studies the adipose tissue was sampled after the diagnosis, rendering results difficult to interpret because treatment may affect subsequent organochlorine concentrations (Gammon et al. 1996; Morgan and Roan 1971).

In the present study, we investigated the hypothesis that organochlorines are related to an increased NHL risk, and we are the first to use adipose tissue sampled before the NHL diagnosis to assess organochlorine exposures in a population-based setting.

## Materials and Methods

Between December 1993 and May 1997, 160,725 persons 50–64 years of age were invited to participate in the prospective Diet, Cancer and Health study. The participants had to be born in Denmark, live in Copenhagen or Aarhus, and be cancer free at the time of invitation. A total of 57,053 persons of those invited were enrolled in the cohort. Upon enrollment, each participant completed self-administered questionnaires on dietary habits, health status, family history of cancer, social factors, reproductive factors, and lifestyle habits. Participation was based on written informed consent. Staff members in the study clinics obtained height and weight measurements and took a buttock adipose tissue biopsy from each participant, yielding an average of 29 mg (range, 1–97 mg) tissue. All samples were frozen at −20°C within 2 hr and put in liquid nitrogen vapor (maximum temperature, −150°C) for long-term storage within 8 hr of collection. The study was approved by regional ethics committees for Copenhagen and Aarhus.

Using the unique personal identification number allocated to every Danish citizen, we linked all cohort members to the Danish Central Population Registry to obtain information on vital status, disappearance, and emigration. Follow-up for NHL started on the date of enrollment at one of the study clinics (baseline) and continued until date of diagnosis of any cancer (except nonmelanoma skin cancer), date of death, date of emigration, or 31 July 2008 (end of follow-up), whichever came first. The mean and maximum follow-up times were 9.6 and 15 years, respectively. Cancers diagnosed during follow-up were identified by linkage to the nationwide Danish Cancer Registry using the personal identification number.

We excluded 9 of the identified NHL cases because of the diagnosis of another previous cancer, resulting in 278 cases (152 men, 126 women) with NHL. Stored adipose tissue was missing for 22 cases, which were therefore excluded. We used a case–cohort design within the Diet, Cancer and Health cohort. A subcohort consisting of 256 persons was randomly selected among the entire cohort, regardless of case status. Of the final 512 participants, we excluded 17 cases and 11 subcohort persons because of either insufficient availability of adipose tissue for organochlorine analysis or laboratory analysis errors, leaving 239 cases (126 men, 113 women) and 245 subcohort persons (126 men, 119 women).

*Organochlorine analyses.* The organochlorines measured were 10 PCB congeners (congeners 99, 118, 138, 153, 156, 170, 180, 183, 187, and 201) and 8 pesticides analytes [*p*,*p*´-DDT, *p*,*p*´-dichlorodiphenyldichloroethylene (*p*,*p*´-DDE), β-hexachlorocyclohexane, dieldrin, hexachlorobenzene, *cis*-nonachlor, *trans*-nonachlor, and oxychlordane].

Samples were analyzed at the Centre de toxicologie du Québec, Institut National de Santé Publique du Québec Quebec City, Quebec, Canada. Laboratory personnel were blind to the case status. The laboratory is accredited under ISO 17025 by the Canadian Standards Council and participates in many quality control programs, including the Northern Contaminants Program of the Ministry of the Environment of Ontario, the U.K. National External Quality Assessment Scheme, and the German External Quality Assessment Scheme.

The sample was aspirated from the needle into a vacuum-based collection vial. The tissue samples were then fortified with internal standards, mixed with dichloromethane, and chemically dried using sodium sulfate. Part of the organic solvent was used to determine the percentage of total lipids in the sample. The remaining fraction was concentrated by evaporation, and purified using gel permeation chromatography, and cleaned up on a Florisil column (Agilent Technologies, Mississauga, Ontario, Canada). The extracts were analyzed on a gas chromatography/mass spectroscopy instrument (model 6890/5973; Agilent Technologies) using a DB-XLB capillary column [60 m long, 0.25 mm inner diameter and 0.25 μm film thickness (Agilent Technologies)]. The measurement of ions generated after negative chemical ionization was performed in selective ion mode. The concentration calculations took the recovery of isotopically labeled internal standards into account. For additional information on instrumentation and analytical protocols, see Supplemental Material, page S2 (http://dx.doi.org/10.1289/ehp.1103573).

We determined the total lipid content of the designated extract using a gravimetric method. A 200 μL sample was precisely weighed on an analytic balance, and the solvent was evaporated at room temperature. The resulting lipid weight was adjusted to the initial sample weight, and the percentage of lipid content was calculated. The organochlorine concentrations were expressed in microgram per kilogram of lipids.

For each of the analytes, the limit of detection (LOD) was determined by first estimating the concentration equivalent to a signal:noise ratio of 3. We then measured 10 replicates of a sample with the analyte at concentrations 4–10 times the estimated LOD and defined the final LOD as three times the standard deviation of the 10 replicates. The organochlorines could be divided into four groups according to LOD. Group 1 was PCB-99; group 2, *p*,*p*´-DDE, *p*,*p*´-DDT, and β-hexachlorocyclohexane; group 3, hexachlorobenzene; and group 4, all other compounds. The LOD was 1.00, 0.30, 0.20, and 0.10 μg/kg for groups 1, 2, 3, and 4, respectively. For each study sample, the LOD was adjusted according to the weight and lipid content of the sample, resulting in a wide range of sample-specific LODs for each compound.

Routine checks of the accuracy and precision of the organochlorine measurements were done using reference materials from the National Institute of Standards and Technology (Gaithersburg, MD, USA) and by participating in quality assessment schemes. The interday precision was 5.1–7.3% for the PCB congeners and 5.0–10.5% for the pesticides. Based on spiked levels (5 μg/kg in corn oil, *n* = 3), recovery was 87–96% for the different PCB congeners and 70–94% for the organochlorine pesticides. For details concerning analytical quality assurance/control and individual recoveries, see Supplemental Material, page S2 and Table S1 (http://dx.doi.org/10.1289/ehp.1103573).

*Statistical methods.* In accordance with the sampling design, the incidence rate ratios (IRRs) for NHL were estimated by a Cox proportional hazard model (PHREG in SAS, version 9.1; SAS Institute Inc., Cary, NC, USA).

The two-sided 95% confidence interval (CI) and *p*-values were based on the robust estimates of the variance-covariance matrix (Barlow 1994) and the Wald’s test statistic for regression parameters in Cox regression models. All statistical tests were two sided, and statistical significance was defined as *p* < 0.05. Age was the time scale, ensuring the risk estimate was based on comparisons of individuals at the same age, thus adjusting for age (Thiebaut and Benichou 2004), and analyses were stratified according to sex.

Based on the exposure distribution among all participants with levels above the LOD, we generated five exposure groups (0th–25th, 25th–50th, 50th–75th, 75th–90th, and > 90th percentiles) for each compound. Cutoff percentiles were based on exposure distribution among all participants because we hypothesized that exposure increased the risk for NHL. Thus, basing cutoff percentiles on the exposure of the subcohort alone may have resulted in relatively few cases in the lowest exposure category (reference group), possibly compromising statistical power when estimating IRRs for the upper exposure groups.

Relative risk was estimated by contrasting each of the upper exposure groups with the lowest exposure group. Case and subcohort members with organochlorine levels below the LOD were excluded instead of being assigned a value based on the LOD, as the LOD varied substantially among study samples because of differences in lipid content. We investigated the sensitivity of the results relating to this exclusion by repeating categorical analyses with persons who had organochlorine levels below their sample-specific LOD assigned as follows: If the LOD for their adipose sample was below the 25th percentile of the distribution of measured concentrations (based on all samples with values > LOD), the subject was assigned to the lowest exposure category. If the LOD for their sample was above the 25th percentile of measured concentrations, they were assigned to a separate exposure group (modeled as a dummy variable). For linear analyses (of continuous exposures), the concentration for subjects with sample LODs below the 25th percentile was imputed as the specific LOD for the sample, whereas those with sample LODs above the 25th percentile were included in a separate exposure group, as above. This strategy assumes no (categorical analyses) or low (linear analyses) exposure misclassification.

Analyses were conducted for individual organochlorines, as well as for the sum of PCB congeners, chlordanes (*cis*- and *trans*-nonachlor and the metabolite oxychlordane), and DDTs (DDT and DDE). In one of the analyses, we grouped PCBs according to immunotoxicological properties (PCB congeners 138, 153, and 180), as previously suggested (Van Den Heuvel et al. 2002). We evaluated the associations between each organochlorine and NHL risk using linear splines: first with three boundaries (25th, 50th, and 75th percentiles) and then with nine boundaries placed at the 10th, 20th, 30th . . . 90th percentiles among all participants. The linearity was evaluated graphically in addition to being evaluated with a numerical test using the likelihood ratio test statistic to compare the model assuming linearity with the linear spline model. Because none of the organochlorines deviated from linearity (data not shown), we calculated IRR per interquartile range (IQR) based on the continuous organochlorine concentrations. The IRRs for NHL in association with concentrations of individual organochlorines in adipose tissue were calculated with and without adjusting for body mass index (BMI) as a continuous variable. Obesity has previously been associated with NHL (Skibola 2007), and a high BMI could influence the adipose tissue concentration of the organochlorine compounds. We estimated the linear effect of exposure for participants below and above the median BMI (26.0 kg/m^2^) among all participants and tested possible interactions by comparing these linear effect estimates.

The potential sex-linked modification of the relationship between NHL risk and exposure to DDT, *cis*-nonachlor, and oxychlordane was also evaluated by introducing interaction terms into the model and testing by the Wald test. Finally, analyses were stratified by the time from adipose sampling to disease diagnosis for DDT, *cis*-nonachlor, and oxychlordane because stronger associations between NHL and organochlorine levels were previously reported in early (< median) compared with late (≥ median) follow-up periods in a study of three unrelated cohorts (Engel et al. 2007).

Relationships between the different lipid-adjusted PCB congeners and pesticide analytes detected over LOD were assessed among noncases by the Spearman rank correlation.

## Results

At baseline, cases were older (median, 64 years of age; range, 51–77 years) than subcohort persons (median, 56 years of age; range, 50–65 years), 53% of cases and 46% of subcohort persons were obese (BMI ≥ 26), and sex distribution among cases (47% female) and subcohort persons (49% female) was similar. Organochlorines were quantified in most adipose tissue samples for *p*,*p*´-DDE, β-hexachlorocyclohexane, *trans*-nonachlor, hexachlorobenzene, and PCB congeners and in at least 56% of samples for the remaining organochlorines (Table 1).

**Table 1:** Organochlorines in adipose tissue from case and subcohort members.

IQR (all; μg/kg lipid)	Above LOD [*n* (%)]			Median (5th–95th percentile; μg/kg lipid)		
	Compound	Cases	Subcohort	Cases	Subcohort
*p*,*p*´-DDT		21		137 (57.3)		137 (55.9)		24 (9–83)		21 (10–58)
*p*,*p*´-DDE		710		238 (99.6)		237 (96.7)		700 (180–2,400)		640 (170–2,300)
β-Hexachlorocyclohexane		32		225 (94.1)		220 (89.8)		59 (31–110)		56 (33–115)
*cis*-Nonachlor		4.7		155 (64.9)		156 (63.7)		8.1 (3.5–17.0)		6.8 (3.1–16.0)
*trans*-Nonachlor		21		235 (98.3)		232 (94.7)		35 (17–76)		32 (16–71)
Oxychlordane		14		169 (70.7)		171 (69.8)		26 (15–47)		23 (12–45)
Dieldrin		16		140 (58.6)		144 (59.0)		24 (9–49)		18 (8–49)
Hexachlorobenzene		35		204 (85.4)		213 (86.9)		54 (20–120)		52 (19–110)
PCB-118		23		233 (97.5)		233 (95.1)		34 (16–70)		35 (17–72)
PCB-156		13		235 (98.3)		236 (96.3)		34 (20–61)		34 (21–57)
PCB-99		16.5		171 (71.5)		177 (72.2)		28 (14–57)		26 (12–53)
PCB-138		80		238 (99.6)		243 (99.2)		150 (57–270)		140 (66–260)
PCB-153		130		239 (100.0)		244 (99.6)		300 (170–470)		300 (160–510)
PCB-170		43		238 (99.6)		243 (99.2)		100 (65–170)		100 (68–170)
PCB-180		70		239 (100.0)		245 (100.0)		200 (120–340)		200 (130–320)
PCB-183		12		226 (94.6)		227 (92.7)		25 (11–40)		24 (12–47)
PCB-187		22		238 (99.6)		240 (98.0)		58 (29–92)		56 (30–99)
PCB-201		8		224 (93.7)		227 (92.7)		19 (11–33)		19 (11–31)

When controlling for age, sex, and BMI, we found suggestions of a monotonic dose–response relationship in association with concentrations of DDT and *cis*-nonachlor, with respective IRRs for the highest versus lowest exposure categories of 1.64 (95% CI: 0.68, 3.96) and 2.52 (95% CI: 1.04, 6.09; Table 2). In the linear analyses, IRRs for IQR increases in exposure to DDT, *cis*-nonachlor and oxychlordane were 1.35 (95% CI: 1.10, 1.66), 1.13 (95% CI: 0.94, 1.36), and 1.11 (95% CI: 0.89, 1.38), respectively (Table 2). Estimates with and without adjusting for BMI (in addition to controlling for sex and age) were similar in both the linear and categorical analyses (Table 2). BMI did not significantly modify the effect of any of the organochlorines, and *p*-values for the interaction were ≥ 0.16. Furthermore, the effects among obese persons (BMI ≥ 26) were not consistent for all the organochlorines, with enhanced effects observed for some and decreased effects for others (data not shown). We found no significant association or consistent trend between NHL risk and adipose concentrations of the five other pesticides or the chlordanes and DDT sums (Table 2), nor did we find any linear risk pattern for any of the individual PCB congeners or their sums (Table 3). We found higher relative risk estimates among men than women in association with DDT, *cis*-nonachlor, and oxychlordane (Table 4).

**Table 2:** IRRs of NHL in association with adipose tissue concentrations of organochlorine pesticides.

Compound/concentration*a* (μg/kg lipids)	*n *(cases/subcohort)	IRR (95% CI)		
		Model 1*b*	Model 2*c*
*p*,*p*´-DDT						
6–15		29/37		1.00		1.00
15–22		32/32		1.09 (0.54, 2.18)		1.06 (0.52, 2.14)
22–36		35/38		1.12 (0.57, 2.19)		1.03 (0.51, 2.09)
36–49		23/19		1.25 (0.55, 2.80)		1.21 (0.53, 2.75)
49–460		18/11		1.73 (0.72, 4.14)		1.64 (0.68, 3.96)
Linear estimate per IQR*d*		137/137		1.36 (1.11, 1.68)		1.35 (1.10, 1.66)
*p*,*p*´-DDE						
68–390		59/58		1.00		1.00
390–680		53/64		0.82 (0.49, 1.37)		0.83 (0.49, 1.39)
680–1,100		63/57		1.03 (0.62, 1.72)		1.04 (0.62, 1.73)
1,100–1,700		34/36		0.82 (0.45, 1.50)		0.79 (0.43, 1.46)
1,700–8,000		29/22		1.12 (0.58, 2.17)		1.10 (0.57, 2.14)
Linear estimate per IQR*d*		238/237		1.03 (0.86, 1.22)		1.02 (0.86, 1.22)
β-Hexachlorocyclohexane						
15–45		50/60		1.00		1.00
45–57		50/54		1.15 (0.67, 1.99)		1.15 (0.66, 2.00)
57–77		61/58		1.27 (0.74, 2.17)		1.24 (0.72, 2.15)
77–96		38/25		1.59 (0.83, 3.03)		1.49 (0.76, 2.92)
96–370		26/23		1.06 (0.53, 2.11)		1.02 (0.50, 2.07)
Linear estimate per IQR*d*		225/220		0.99 (0.83, 1.17)		0.96 (0.81, 1.15)
*cis*-Nonachlor						
3–5		30/50		1.00		1.00
5–7		35/42		1.23 (0.64, 2.35)		1.19 (0.61, 2.30)
7–10		42/32		1.56 (0.82, 2.96)		1.53 (0.80, 2.91)
10–15		27/21		1.64 (0.79, 3.41)		1.76 (0.84, 3.67)
15–39		21/11		2.60 (1.08, 6.27)		2.52 (1.04, 6.09)
Linear estimate per IQR*d*		155/156		1.13 (0.94, 1.36)		1.13 (0.94, 1.36)
*trans*-Nonachlor						
6–25		47/67		1.00		1.00
25–33		53/52		1.33 (0.78, 2.28)		1.39 (0.81, 2.39)
33–46		65/60		1.40 (0.84, 2.36)		1.43 (0.85, 2.40)
46–62		41/33		1.37 (0.75, 2.52)		1.38 (0.75, 2.54)
62–290		29/20		1.60 (0.79, 3.24)		1.54 (0.76, 3.14)
Linear estimate per IQR*d*		235/232		1.03 (0.90, 1.18)		1.01 (0.89, 1.16)
Compound/concentration*a* (μg/kg lipids)		*n *(cases/subcohort)		IRR (95% CI)		
				Model 1*b*		Model 2*c*
Oxychlordane						
8–19		30/52		1.00		1.00
19–25		38/46		1.31 (0.71, 2.43)		1.33 (0.72, 2.46)
25–33		46/39		1.87 (1.00, 3.49)		1.86 (1.00, 3.47)
33–41		34/18		2.81 (1.28, 6.14)		2.69 (1.22, 5.96)
41–354		21/16		1.95 (0.85, 4.46)		1.92 (0.84, 4.41)
Linear estimate per IQR*d*		169/171		1.13 (0.92, 1.40)		1.11 (0.89, 1.38)
Dieldrin						
6–13		33/32		1.00		1.00
13–20		21/52		0.38 (0.19, 0.76)		0.36 (0.17, 0.73)
20–29		35/37		0.95 (0.49, 1.85)		0.91 (0.46, 1.79)
29–41		22/12		2.05 (0.92, 4.54)		1.94 (0.87, 4.34)
41–350		18/11		1.42 (0.60, 3.36)		1.27 (0.52, 3.12)
Linear estimate per IQR*d*		140/144		1.03 (0.94, 1.14)		1.03 (0.94, 1.13)
Hexachlorobenzene						
9–37		47/56		1.00		1.00
37–53		50/51		1.08 (0.62, 1.88)		1.07 (0.61, 1.87)
53–72		45/56		0.84 (0.48, 1.49)		0.77 (0.42, 1.43)
72–98		37/33		1.15 (0.60, 2.18)		1.07 (0.55, 2.09)
98–360		25/17		1.25 (0.57, 2.72)		1.16 (0.53, 2.58)
Linear estimate per IQR*d*		204/213		1.00 (0.82, 1.21)		0.98 (0.80, 1.19)
ΣChlordanes*e*						
9–37		55/59		1.00		1.00
37–58		51/68		0.77 (0.46, 1.30)		0.75 (0.44, 1.27)
58–83		64/53		1.18 (0.70, 1.99)		1.10 (0.64, 1.91)
83–108		35/35		0.84 (0.45, 1.55)		0.80 (0.43, 1.50)
108–439		30/17		1.41 (0.68, 2.94)		1.32 (0.63, 2.79)
Linear estimate per IQR*f*		235/232		1.04 (0.86, 1.26)		1.02 (0.84, 1.24)
ΣDDTs*g*						
76–390		57/56		1.00		1.00
390–690		57/67		0.86 (0.51, 1.43)		0.87 (0.52, 1.47)
690–1,100		61/56		1.02 (0.61, 1.72)		1.03 (0.61, 1.74)
1,100–1,714		36/37		0.86 (0.48, 1.56)		0.83 (0.46, 1.52)
1,714–8,041		27/21		1.09 (0.56, 2.15)		1.08 (0.55, 2.14)
Linear estimate per IQR*h*		238/237		1.03 (0.87, 1.23)		1.03 (0.86, 1.23)

**Table 3:** IRRs for NHL and 95% CI in association with adipose tissue concentrations of PCBs.

Compound/concentration*a* (μg/kg lipids)	*n* (cases/subcohort)	IRR (95% CI)		
		Model 1*b*	Model 2*c*
PCB-118						
10–25		53/50		1.00		1.00
25–34		63/62		0.97 (0.57, 1.66)		0.88 (0.50, 1.56)
34–48		58/56		1.00 (0.59, 1.71)		0.96 (0.55, 1.65)
48–62		34/38		0.78 (0.42, 1.45)		0.67 (0.34, 1.31)
62–150		25/27		0.79 (0.40, 1.55)		0.72 (0.36, 1.44)
Linear estimate per IQR*d*		233/233		0.92 (0.72, 1.17)		0.88 (0.68, 1.14)
PCB-156						
13–28		62/53		1.00		1.00
28–34		51/63		0.56 (0.33, 0.96)		0.59 (0.34, 1.02)
34–41		54/59		0.64 (0.38, 1.09)		0.68 (0.40, 1.16)
41–50		45/35		0.83 (0.46, 1.50)		0.94 (0.51, 1.75)
50–88		23/26		0.57 (0.28, 1.15)		0.66 (0.31, 1.37)
Linear estimate per IQR*d*		235/236		0.95 (0.75, 1.20)		1.01 (0.79, 1.29)
PCB-99						
9–20		32/49		1.00		1.00
20–27		42/41		1.63 (0.88, 3.02)		1.60 (0.85, 3.01)
27–37		53/44		1.59 (0.87, 2.91)		1.56 (0.84, 2.89)
37–47		24/27		1.20 (0.58, 2.48)		1.20 (0.58, 2.49)
47–110		20/16		1.45 (0.61, 3.43)		1.42 (0.59, 3.40)
Linear estimate per IQR*d*		171/177		1.10 (0.83, 1.44)		1.09 (0.83, 1.43)
PCB-138						
29–100		53/49		1.00		1.00
100–140		44/69		0.66 (0.38, 1.13)		0.66 (0.38, 1.14)
140–180		74/63		1.05 (0.63, 1.75)		1.04 (0.62, 1.74)
180–230		41/34		1.25 (0.67, 2.31)		1.25 (0.67, 2.33)
230–380		26/28		0.68 (0.34, 1.34)		0.68 (0.34, 1.36)
Linear estimate per IQR*d*		238/243		0.99 (0.78, 1.25)		0.99 (0.78, 1.26)
PCB-153						
98–240		56/55		1.00		1.00
240–300		57/61		0.87 (0.52, 1.47)		0.88 (0.52, 1.50)
300–370		56/73		0.64 (0.38, 1.07)		0.67 (0.40, 1.12)
370–430		42/28		1.38 (0.75, 2.53)		1.50 (0.81, 2.78)
430–730		28/27		0.79 (0.39, 1.57)		0.85 (0.42, 1.73)
Linear estimate per IQR*d*		239/244		0.94 (0.75, 1.19)		0.97 (0.77, 1.23)
PCB-170						
37–87		57/61		1.00		1.00
87–100		47/41		1.09 (0.63, 1.89)		1.19 (0.68, 2.09)
100–130		69/78		0.79 (0.48, 1.30)		0.93 (0.54, 1.59)
130–150		42/32		1.16 (0.64, 2.08)		1.46 (0.75, 2.83)
150–230		23/31		0.64 (0.32, 1.29)		0.80 (0.38, 1.69)
Linear estimate per IQR*d*		238/243		0.89 (0.68, 1.18)		0.98 (0.72, 1.33)
Compound/concentration*a* (μg/kg lipids)		*n* (cases/subcohort)		IRR (95% CI)		
				Model 1*b*		Model 2*c*
PCB-180						
71–170		53/60		1.00		1.00
170–200		55/52		0.94 (0.56, 1.67)		1.03 (0.60, 1.77)
200–240		61/54		1.04 (0.62, 1.83)		1.19 (0.69, 2.05)
240–290		49/48		0.98 (0.50, 1.59)		1.09 (0.59, 2.01)
290–480		21/31		0.54 (0.27, 1.08)		0.69 (0.32, 1.46)
Linear estimate per IQR*d*		239/245		0.90 (0.75, 1.08)		0.99 (0.77, 1.27)
PCB-183						
6–19		59/53		1.00		1.00
19–24		35/54		0.59 (0.34, 1.05)		0.58 (0.32, 1.03)
24–31		69/60		0.92 (0.55, 1.53)		0.91 (0.54, 1.51)
31–39		40/34		1.03 (0.56, 1.90)		1.03 (0.56, 1.90)
39–65		23/26		0.66 (0.33, 1.31)		0.68 (0.34, 1.37)
Linear estimate per IQR*d*		226/227		0.87 (0.70, 1.08)		0.88 (0.70, 1.10)
PCB-187						
17–46		61/58		1.00		1.00
46–56		49/62		0.68 (0.40, 1.15)		0.69 (0.40, 1.17)
56–68		62/60		0.90 (0.54, 1.51)		0.97 (0.57, 1.64)
68–84		44/33		1.12 (0.60, 2.07)		1.30 (0.68, 2.47)
84–140		22/27		0.62 (0.30, 1.27)		0.69 (0.33, 1.44)
Linear estimate per IQR*d*		238/240		0.88 (0.71, 1.11)		0.92 (0.73, 1.15)
PCB-201						
6–15		43/47		1.00		1.00
15–19		62/61		0.91 (0.52, 1.59)		0.98 (0.56, 1.73)
19–23		58/47		1.10 (0.62, 1.97)		1.20 (0.66, 2.21)
23–28		36/44		0.69 (0.37, 1.29)		0.82 (0.41, 1.67)
28–45		25/28		0.73 (0.34, 1.56)		0.88 (0.38, 2.03)
Linear estimate per IQR*d*		224/227		0.85 (0.64, 1.13)		0.93 (0.68, 1.28)
ΣPCB*e*						
150–770		62/59		1.00		1.00
770–939		55/66		0.74 (0.45, 1.24)		0.74 (0.44, 1.24)
939–1,143		57/62		0.78 (0.47, 1.30)		0.81 (0.48, 1.35)
1,143–1,351		42/32		1.08 (0.59, 1.95)		1.15 (0.63, 2.11)
1,351–2,157		23/26		0.65 (0.32, 1.33)		0.71 (0.34, 1.45)
Linear estimate per IQR*f*		239/245		0.95 (0.76, 1.19)		0.99 (0.79, 1.25)
ΣPCB_immune_*g*						
150–530		60/58		1.00		1.00
530–650		55/63		0.77 (0.46, 1.30)		0.79 (0.47, 1.34)
650–790		59/65		0.77 (0.47, 1.29)		0.82 (0.49, 1.38)
790–930		42/32		1.15 (0.64, 2.09)		1.29 (0.70, 2.38)
930–1,490		23/27		0.62 (0.31, 1.26)		0.68 (0.33, 1.41)
Linear estimate per IQR*h*		239/245		0.95 (0.76, 1.18)		0.99 (0.78, 1.24)

**Table 4:** IRRs for NHL and 95% CI in association with adipose concentrations of *p*,*p´*-DDT, *cis*-nonachlor, and oxychlordane within strata of sex.

Compound/concentration*a* (μg/kg lipids)	*n* (cases/subcohort)			IRR (95% CI)			*p*-Value for difference*b*
	Men	Women	Men	Women			
*p*,*p*´-DDT										
6–15		8/16		21/21		1.00		1.00		
15–22		16/13		16/19		1.90 (0.60, 6.02)		0.77 (0.31, 1.89)		
22–36		20/11		15/27		3.36 (1.06, 10.64)		0.48 (0.20, 1.17)		
36–49		10/7		13/12		2.05 (0.53, 7.87)		0.88 (0.31, 2.47)		
49–460		9/7		9/4		2.30 (0.60, 8.77)		1.69 (0.47, 6.08)		
Linear estimate per IQR*c*		63/54		74/83		1.50 (1.14, 1.98)		1.15 (0.72, 1.86)		0.35
*cis*-Nonachlor										
3–5		7/14		23/31		1.00		1.00		
5–7		10/21		25/21		1.02 (0.30, 3.47)		1.37 (0.60, 3.12)		
7–10		19/20		23/17		2.00 (0.61, 6.51)		1.41 (0.59, 3.34)		
10–15		17/11		10/10		3.00 (0.86, 10.51)		1.24 (0.45, 3.44)		
15–39		19/8		2/3		4.42 (1.21, 16.09)		0.67 (0.10, 4.46)		
Linear estimate per IQR*c*		72/74		83/82		1.39 (1.00, 1.93)		0.94 (0.73, 1.20)		0.06
Oxychlordane										
8–19		14/24		16/28		1.00		1.00		
19–25		17/23		21/23		1.29 (0.52, 3.22)		1.44 (0.62, 3.37)		
25–33		18/15		28/24		2.05 (0.78, 5.35)		1.74 (0.76, 3.97)		
33–41		13/3		21/15		6.15 (1.46, 25.93)		1.81 (0.71, 4.66)		
41–354		12/6		9/10		3.45 (0.97, 12.20)		1.18 (0.39, 3.52)		
Linear estimate per IQR*c*		74/71		95/100		1.27 (0.90, 1.79)		0.97 (0.73, 1.29)		0.23
**a**The cutoffs between exposure groups were 25th, 50th, 75th, and 90th percentiles. **b**For Wald test for interaction. **c**Linear estimate per IQR based on pesticide concentrations analyzed as continuous variables.										

The correlation between the organochlorine pesticides varied, with generally low correlations for dieldrin, hexachlorobenzene, and the other pesticides [*r*_Spearman_ (*r*_S_) mostly < 0.30], strong correlations between the chlordanes (*r*_S_ = 0.74–0.91), and moderate correlation for the other pesticides (*r*_S_ = 0.30–0.50). The PCBs were moderately to highly correlated with each other [*r*_S_ = 0.25–0.98; for a complete listing of correlation coefficients, see Supplemental Material, Table S2, (http://dx.doi.org/10.1289/ehp.1103573)].

We found no significant difference in NHL risk among persons diagnosed in early follow-up (< 9.6 years after baseline) compared with those diagnosed in late follow-up [≥ 9.6 years after baseline; see Supplemental Material, Table S3 (http://dx.doi.org/10.1289/ehp.1103573)]. Sensitivity analyses assigning persons with organochlorine levels below the LOD to the lowest exposure category (if the LOD for the actual sample was below the 25th percentile) or to a separate exposure category (for the remaining samples below the LOD) showed results similar to those from analyses excluding samples below the LOD (see Supplemental Material, Table S4).

## Discussion

In this study, we found that lipid concentrations of DDT, *cis*-nonachlor, and oxychlordane in a general Danish population were associated with elevated risk of NHL. A positive monotonic dose–response relationship was suggested for DDT and *cis*-nonachlor, but the positive trend was less consistent for oxychlordane. The estimated effect of all three compounds was strongest among men. We observed no clear or consistent evidence of associations between NHL and adipose concentrations of PCB congeners or the other pesticides included in this study.

These data support the hypothesis of a link between DDT and the risk of NHL as suggested by some early occupational studies (Baris et al. 1998; Cantor et al. 1992; Woods et al. 1987). However, most previous studies reported no association (De Roos et al. 2003, 2005; Hardell and Eriksson 1999; Rothman et al. 1997; Spinelli et al. 2007), including three with exposures measured in blood samples. In light of the high level of correlation between DDT measured in adipose and blood (Archibeque-Engle et al. 1997; Stellman et al. 1998; Whitcomb et al. 2005), we would expect a degree of interchangeability and little discrepancy in health results pertaining to this compound. We also found no association between DDE and NHL risk, which could be expected because DDE is more stable than DDT, was detected at levels over LOD in > 97% of adipose samples, and may therefore represent a more accurate biomarker of exposure than DDT. Thus, we cannot exclude the possibility that the observed association with DDT is a false-positive result.

Results for individual chlordanes are limited, and only four previous studies have considered the associations between NHL and chlordane isomers or the metabolite oxychlordane (De Roos et al. 2005; Hardell et al. 2001; Quintana et al. 2004; Spinelli et al. 2007). Results are mixed: one group found an association with oxychlordane but not *cis*-nonachlor (Spinelli et al. 2007), whereas another found an association with *cis*-nonachlor but not oxychlordane (Hardell et al. 2001). Two other groups studied only oxychlordane; one found results in accord with ours (Quintana et al. 2004), whereas the other found no association (De Roos et al. 2005). The persistence, bioaccumulation, and distribution in human tissue as well as toxic effects of each individual chlordane depend on its chemical structure, so we cannot compare our results for *cis*-nonachlor and oxychlordane with the results of studies that considered the sum of chlordanes only (Cantor et al. 2003; De Roos et al. 2003; McDuffie et al. 2001).

We observed no association between NHL and PCBs, in contrast with previous studies that reported associations with PCB concentrations in blood (Bertrand et al. 2010; De Roos et al. 2005; Engel et al. 2007; Hardell et al. 2001; Rothman et al. 1997; Spinelli et al. 2007). The individual congeners used when calculating the PCB sum and the actual individual PCB congeners associated with NHL differed between studies, but three of these studies reported an association between PCB-180 and NHL (Bertrand et al. 2010; De Roos et al. 2005; Spinelli et al. 2007). We detected this congener over LOD in 100% of our adipose samples but did not observe an association with NHL in our study population. Three previous studies have reported no association, in accordance with our findings (Cocco et al. 2008; Laden et al. 2010; Quintana et al. 2004), including one that measured PCBs in adipose tissue samples (Quintana et al. 2004). The choice of biological specimen used in exposure quantification may lead to discrepant human health results among studies. Our study is the first to use prospectively sampled adipose tissue in exposure assessment. Adipose tissue is the principal storage medium for lipophilic organochlorines in the human body (Anderson 1985) and has been regarded by various authors (Allam and Lucena 2001; Hardell et al. 1996; Quintana et al. 2004) as the preferred indicator of human exposure because it represents cumulative internal exposure. Blood samples, which have been used to assess exposure in four previous studies (Cantor et al. 2003; De Roos et al. 2005; Hardell et al. 2001; Spinelli et al. 2007), are more easily obtained and less costly but may represent more recent exposure than levels in adipose samples collected at the same time (Archibeque-Engle et al. 1997).

The limited sample size is a weakness of this study, which limited the power to detect statistically significant interactions with sex and prevented analyses of association for specific NHL subtypes. A better statistical power would have been achieved with a larger sample size (e.g., by selecting a larger subcohort), but organochlorine analysis costs were a constraint. Further, the age distribution between the cases and subcohort was quite different, which is expected as the incidence rate for NHL increases with age, and cases were generally older than the subcohort members. In our analyses, however, we used age as the time scale, which is recommended to avoid bias when analyzing the effect of covariates that correlate with age (Thiebaut and Benichou 2004). Another limitation of this study in relation to some of the pesticides is the number of samples with concentrations below the LOD. It can be argued that the exclusion of these samples in the present study could introduce bias. Thus, we investigated the sensitivity of our results related to assignment of organochlorine levels below the LOD to the lowest exposure group if the LOD for the actual sample was below the 25th percentile (the cutoff between the two lower exposure categories). We found our results robust in these analyses. Retaining samples below the LOD, for example, by imputing LOD/_√_^–^2 as their value, is advisable only in cases in which < 5–10% of samples are below LOD (Lubin et al. 2004). This strategy is problematic in the present study because individual LODs depend on the amount of adipose tissue in that particular sample, such that LOD varied by up to two orders of magnitude between samples below LOD for the same organochlorine. Thus, imputing these LODs might mask or reverse true associations.

Two studies have estimated associations between organochlorines measured in adipose tissue and NHL risk. The first was a small study that examined only summed chlordanes (Hardell et al. 1996) in samples obtained after diagnosis (in cases), whereas the other used adipose samples taken post mortem (Quintana et al. 2004). Prospective studies are advantageous when investigating carcinogenic effects exerted years before clinical presentation, whereas organochlorine levels in adipose samples collected after diagnosis have been shown to be affected by treatment (Gammon et al. 1996) and weight loss (Glynn et al. 2003; Jandacek et al. 2005) and to possibly alter metabolic function (Morgan and Roan 1971; Mullerova and Kopecky 2007), which could be related to weight fluctuations, ultimately affecting organochlorine levels. We sampled adipose tissue up to 15 years before the NHL diagnosis, and given the relatively long half-lives of DDT, *cis*-nonachlor, and oxychlordane (up to 6 years), our prospective measures represent a relatively stable internal dose that is likely to reflect exposures several years before follow-up for cancer started.

We are the first group to report associations between NHL and DDT, *cis*-nonachlor, and oxychlordane mainly (or possibly only) among men. Eight previous studies considered both sexes, but only one investigated sex-specific effects of DDT on NHL (Miligi et al. 2003) and reported no association for either sex. The stronger associations among men in our study population may be due to differences in metabolism and elimination of the compounds (e.g., women can excrete these compounds via breast-feeding).

Some studies have suggested that BMI could confound associations between organochlorine concentrations and NHL, although this is controversial (Cox et al. 1999). A positive association between high BMI and organochlorines may be expected in relation to prolonged consumption of fatty foods, which are often associated with overweight and possibly a high intake of lipophilic compounds; conversely, however, a high BMI and body fat content could result in a dilution effect. We investigated whether persons with a high BMI had greater risk of NHL than persons with low BMI, analyzing data both with and without adjusting for BMI, and found no evidence of effect modification or confounding. BMI was measured at the time of enrollment/adipose sampling, which is relevant for assessing the effect of BMI on the adipose sample but does not reflect effects of BMI over time.

The timing of diagnosis relative to the organochlorine measurement has been investigated in only one previous study (Engel et al. 2007), which reported a stronger exposure–response association among subjects diagnosed within the years before median follow-up (early follow-up) compared with subjects diagnosed in the years after median follow-up (late follow-up). We observed no significant differences related to the duration of follow-up and thus could not confirm this result.

The major strength of this study is the prospective collection of adipose tissue samples in a general population setting. Follow-up was virtually complete, and reliable nationwide registries provided information on vital status and cancer. Selection bias is unlikely because cases and subcohort participants came from the same cohort and fulfilled the same eligibility criteria. Further, recall bias was avoided by the objective exposure assessment method, and disease status could not have biased exposure assessment because adipose tissue was obtained and frozen before cancer diagnosis.

The exact mechanisms by which organochlorines might increase NHL risk remain to be elucidated but the involvement of immunosuppression is suspected. Evidence of the immunotoxicity of organochlorines (including DDT and chlordane) has been reviewed, and both human and toxicological evidence suggests immunotoxicity (Repetto and Baliga 1997). More recent animal and *in vitro* studies confirm this for DDT and individual chlordanes (Reed et al. 2004; Tryphonas et al. 2003); however, this evidence is composed mainly of acute high-dose studies, and the relevance to typical human exposure levels may be questioned. It remains unclear which specific compounds are related to NHL risk and whether this association is causal. We cannot rule out confounding from unaccounted risk factors, for example, other more toxic agents such as furans, dioxins, or coplanar PCBs that are bioaccumulated in parallel with DDT, *cis*-nonachlor, and oxychlordane. More research is needed to clarify possible interactions with organochlorines and other agents such as viruses and genetic polymorphisms to further elucidate biological mechanisms behind this association.

## Conclusion

Our results add new evidence of an association between exposure to DDT and chlordanes and NHL at prospectively measured exposure levels experienced by a general population but do not contribute to the evidence that exposure to PCB congeners may increase the risk of NHL.

## Supplemental Material

(61 KB) PDF
