# Supplementary material for: A Prospective Study of Organochlorines in Adipose Tissue and Risk of Non-Hodgkin Lymphoma
Source: Environ Health Perspect. 2011 Aug 26;120(1):105–11. doi: 10.1289/ehp.1103573 (PMC3261942; doi:10.1289/ehp.1103573)
Supplement: (61 KB) PDF [file ehp.1103573.s001.pdf]

Supporting information for:

A Prospective Study of Organochlorines in Adipose Tissue and Risk of non-Hodgkin's Lymphoma

Elvira Vaclavik Bräuner[1], Mette Sørensen[1], Eric Gaudreau[2], Alain LeBlanc[2], Kirsten Thorup Eriksen[1], Anne Tjønneland[1], Kim Overvad[3] and Ole Raaschou-Nielsen[1]

[1]Institute of Cancer Epidemiology, Danish Cancer Society, 2100 Copenhagen, Denmark

[2]Centre de toxicology du Quebec, Institut National de Santé Publique du Québec, Québec, Canada

[3]Department of Epidemiology, School of Public Health, Aarhus University, Aarhus, Denmark

None of the authors has any potential competing financial interests

Number of Supporting Information pages (including this title page): 7

Table of contents:

Methods S2

Extraction and gas chromatography-mass spectroscopy S2

Quality Assurance/Quality Control of PCB Analyses S2

List of Tables:

Table S1: Recovery, intra-day and inter-day precision for the individual organochlorines S4

Table S2: Spearmann rank correlation between the adipose tissue concentrations of individual PCB congeners and organochlorine pesticide analytes S5

Table S3: Incidence rate ratio for non-Hodgkin lymphoma and 95% confidence interval (CI) in association with adipose concentrations of *p,p´*-DDT, *cis*-nonachlor and oxychlordane within the early and late years of follow-up S6

Table S4: Sensitivity analysis, crude incidence rate ratio for non-Hodgkin lymphoma and 95 % confidence interval (CI) in association with adipose concentrations of DDT, *cis*-Nonachlor and oxychlordane including samples under LOD S7

Methods

Extraction and gas chromatography-mass spectroscopy

The extracts were analyzed on a gas chromatography-mass spectroscopy instrument from (Agilent Technologies (Hewlett-Packard; Palo Alto, CA) model 6890/5973) using a DB-XLB capillary column. (Agilent Technologies; 60 m long, 0.25 mm inner diameter and 0.25 µm film thickness). The measurement of ions generated after negative chemical ionization was performed in selective ion mode. Peak areas were calculated relative to labeled internal standards (PCB 141-$^{13}C_{12}$, PCB 153-$^{13}C_{12}$, PCB 180-$^{13}C_{12}$, hexachlorobenzene-$^{13}C_{6}$, α-hexachlorohexane-$^{13}C_{6}$, p,p'-DDE-$^{13}C_{12}$, oxychlordane-$^{13}C_{10}$, *trans*-nonachlor-$^{13}C_{10}$ and endrin-$^{13}C_{12}$). Samples (3 µl) were injected in the pulsed splitless mode. The temperature program was as follows: 2 min at 100 °C followed by an increase to 200 °C at a rate of 20 °C min^{-1}, increase to 245 °C at a rate of 1.5 °C min^{-1} hold 10 minutes, increase to 280 °C at a rate of 20 °C min^{-1} hold 5 minutes and finally an increase to 330 °C at a rate of 30 °C min^{-1} hold 15 minutes. The total run time was 70.42 minutes. The linearity of the six-point curve was evaluated during the validation of the analytical method, but due to time consumption considerations and to maintain a high throughput production, the quantification was based on a single mid-point calibration.

Quality Assurance/Quality Control of PCB Analyses

Determination of analytical uncertainties found in adipose samples is crucial for the interpretation of data. In this study, each batch consisted of 16 samples, one calibration standard, one procedural blank and one sample of internal reference material. The internal reference material was cod liver oil containing all organochlorines analyzed, and was provided by the National Institute of Standards & Technology (Gaithersburg, MD). The results of the analyses of the reference materials were used to validate the methods on a routine basis. When unacceptably high deviations were obtained from the certified values, the batches concerned were reanalysed. The variation obtained for all compounds during the analysis of all samples was within 15% of the certified values except for DDT which was within 20% of the certified values. Precision was monitored by plotting the results of the internal reference material in control charts with warning and action limits (2 and 3 times the standard deviation of the target value, respectively). These control charts do not, however, indicate the real uncertainty of the methods. Thus, the overall quality and accuracy of the analyses was monitored by regular participation in the Northern Contaminants Program of the Ministry of the Environment of Ontario, the External Quality Assessment Scheme, QUASIMEME as well as the German External Quality Assessment Scheme

for Biological Monitoring in Occupational and Environmental Medicine. For example in QUASIMEME, two samples of tissues with different concentration levels are analysed using routine methods, and the results obtained reported to the organiser. When all participant laboratories had reported their results, an assigned value was determined for each determinant and matrix type, usually by a combination of robust statistics and the use of data from expert laboratories. Where a determinant was present at low concentration and no assigned value could be set, the concentrations were given as indicative values. The z-scores were determined for each compound and when the z-score was less than 2, the determinant was considered satisfactory. If the z-score was between 2 and 3, the determinant was considered questionable and if it was greater than 3, the determinant was unsatisfactory. Results of the inter-comparison exercises mentioned for our laboratory were all deemed satisfactory (the best score).

Table S1: Recovery, intra-day and inter-day precision for the individual organochlorines

Compound	Recovery (%)	Intra-day precision (%)	Inter-day precision (%)
PCB 99	87	4.3	5.0
PCB 118	91	2.9	5.0
PCB 138	94	2.3	7.3
PCB 153	93	2.2	5.0
PCB 156	94	5.0	8.7
PCB 170	95	2.9	5.0
PCB 180	95	2.2	5.1
PCB 183	94	1.3	5.0
PCB 187	94	1.7	5.0
PCB 201	96	3.2	6.1
p,p'-DDT	94	4.2	10.5
p,p'-DDE	88	3.0	5.0
ß-Hexachlorocyclohexane	81	4.1	7.8
cis-Nonachlor	94	2.6	5.4
trans-Nonachlor	89	3.6	5.9
Oxychlordane	82	4.1	9.1
Dieldrin	75	4.7	8.0
Hexachlorobenzene	70	1.6	5.0

Table S2. Spearmann rank correlation between the adipose tissue concentrations of individual PCB congeners and organochlorine pesticide analytes measured above LOD in sub-cohort participants with no NHL diagnose

Compound	DDE	Hexachloro-cyclohexane	*c*-Nonachlor	*t*-Nonachlor	Oxy-chlordane	Dieldrin	Hexachloro-benzene	PCB 118	PCB 156	PCB99	PCB138	PCB153	PCB170	PCB180	PCB183	PCB187	PCB201
p,p'-DDT	0.4286	0.5574	0.4565	0.4726	0.4009	0.1526*	0.0698*	0.5649	0.3352	0.5171	0.5036	0.5002	0.3423	0.3654	0.4258	0.4787	0.2958
p,p'-DDE		0.3341	0.2208	0.3494	0.3664	0.0958*	0.2386	0.5114	0.3372	0.5296	0.7117	0.6689	0.3369	0.3738	0.6867	0.5217	0.1807
Hexachlorocyclohexane			0.3392	0.4127	0.5982	0.2232	0.5553	0.4077	0.2538	0.4169	0.3107	0.2946	0.1678	0.1808	0.2328	0.1977	0.0900*
cis-Nonachlor				0.8821	0.7379	0.2446	0.0652*	0.4696	0.4745	0.5857	0.4489	0.4965	0.3965	0.4329	0.3414	0.5486	0.5651
trans-Nonachlor					0.9062	0.2909	0.2282	0.4987	0.3880	0.5891	0.4660	0.4721	0.3143	0.3277	0.3473	0.4404	0.4493
Oxychlordane						0.3025	0.3837	0.5234	0.4494	0.4662	0.4279	0.4589	0.3258	0.3295	0.2954	0.4137	0.3458
Dieldrin							0.1154*	0.3610	0.1174*	0.1786	0.1314*	0.1295*	0.0272*	0.0309*	0.1005*	0.1020*	-0.0302*
Hexachlorobenzene								0.3605	0.1886	0.0942*	0.2356	0.2171	0.1189*	0.1019*	0.1848	0.1415	0.0008*
PCB 118 (*mono-ortho*)									0.4922	0.6147	0.6880	0.6711	0.3939	0.4092	0.5746	0.5694	0.2489
PCB 156 (*mono-ortho*)										0.4783	0.5307	0.7660	0.9178	0.9114	0.4702	0.7591	0.8353
PCB 99 (*di-ortho*)											0.7895	0.7404	0.4629	0.4903	0.7576	0.6353	0.4166
PCB 138 (*di-ortho*)												0.9196	0.5945	0.6078	0.9302	0.8141	0.4041
PCB 153 (*di-ortho*)													0.8114	0.8306	0.8767	0.9241	0.6568
PCB 170 (*di-ortho*)														0.9802	0.5735	0.8077	0.8991
PCB 180 (*di-ortho*)															0.5856	0.8335	0.9053
PCB 183 (*tri-ortho*)																0.7921	0.4194
PCB 187 (*tri-ortho*)																	0.7815

*non-significant correlations

Table S3. Incidence rate ratio for non-Hodgkin lymphoma and 95% confidence interval (CI) in association with adipose concentrations of *p,p´*-DDT, *cis*-nonachlor and oxychlordane within the early (below 9.6 years of follow-up) and late years of follow-up

Compound	Concentration* (µg/kg lipids)	No. of cases/No. of sub-cohort <9.6 yrs follow-up	≥9.6 yrs follow-up	IRR (95% CI) <9.6 yrs follow-up	≥9.6 yrs follow-up	P-difference
p,p'-DDT	6-15	17/3	12/34	1.00	1.00	
	15-22	21/1	11/31	-	1.03(0.33-3.25)	
	22-36	23/4	12/34	2.02(0.50-8.07)	0.80(0.25-2.54)	
	36-49	13/1	10/18	-	1.14(0.32-4.06)	
	49-460	15/2	2/9	1.53(0.26-9.15)	1.84(0.35-9.70)	
	Linear estimate per IQR**	89/11	48/126	1.26(0.85-1.86)	1.43(0.83-2.45)	0.71
cis-Nonachlor	3-5	17/3	13/42	1.00	1.00	
	5-7	27/5	8/37	1.96(0.47-8.17)	0.60(0.19-1.87)	
	7-10	31/1	11/36	-	1.22(0.41-3.57)	
	10-15	16/2	11/19	2.95(0.57-15.3)	1.50(0.48-4.68)	
	15-39	12/2	9/9	3.58(0.64-20.2)	3.69(0.97-14.1)	
	Linear estimate per IQR	103/13	52/143	1.29(0.72-2.29)	1.18(0.93-1.50)	0.78
Oxychlordane	8-19	19/6	11/46	1.00	1.00	
	19-25	26/3	12/43	5.31(1.42-19.9)	2.02(0.58-7.08)	
	25-33	27/4	19/35	5.82(1.47-2.30)	2.31(0.67-7.94)	
	33-41	25/0	9/18	-	1.22(0.30-5.49)	
	41-354	14/2	7/14	1.58(0.29-8.58)	1.30(0.31-5.49)	
	Linear estimate per IQR	111/15	58/156	1.52(0.86-2.69)	0.87(0.61-1.24)	0.10

*The cut-off points between exposure groups were: 25th, 50th, 75th and 90th percentiles

**Linear estimate per inter-quartile range based on pesticide concentrations analyzed as continuous variables

Table S4. Sensitivity analysis, crude incidence rate ratio for non-Hodgkin lymphoma and 95 % confidence interval (CI) in association with adipose concentrations* of DDT, cis-Nonachlor and oxychlordane including samples under LOD**

Compound	Concentration* (µg/kg lipids)	No. of cases/ No. of sub-cohort	Incidence rate ratio (95% CI)**
p,p'-DDT	6-15	49/64	1.00
	15-22	32/32	0.86 (0.46-1.61)
	22-36	35/38	1.01 (0.54-1.88)
	36-49	23/19	1.27 (0.64-2.49)
	49-460	18/11	1.45 (0.62-3.35)
Linear estimate per IQR #		*157/164*	*1.35 (1.11-1.64)*
cis-Nonachlor	3-5	44/69	1.00
	5-7	35/42	0.84 (0.46-1.53)
	7-10	42/32	1.17 (0.64-2.15)
	10-15	27/21	1.30 (0.64-2.63)
	15-39	21/11	2.01 (0.85-4.75)
Linear estimate per IQR		*169/175*	*1.13 (0.94-1.36)*
Oxychlordane	8-19	31/59	1.00
	19-25	38/46	1.89 (1.00-3.55)
	25-33	46/39	2.39 (1.28-4.47)
	33-41	34/18	3.59 (1.63-7.91)
	41-354	21/16	2.35 (0.87-6.38)
Linear estimate per IQR		*170/178*	*1.16 (0.94-1.43)*

*The cut-off points between the exposure groups were the 25th, 50th, 75th and 90th percentiles

**Age as time scale and model stratified according to gender. Participants with OC levels below LOD were assigned to the lowest exposure category if the LOD for the actual sample was below the 25th percentile (i.e., the cutoff point between the two lower exposure categories) because only these belonged for certain to the lowest exposure category). The same samples were included in the linear analyses. A separate dummy 'exposure' variable for the rest of the non-detects with LOD => 25th percentile was included.

#Linear estimate per inter-quartile range based on pesticide concentration analyzed as a continuous variable in a linear model. A separate dummy 'exposure' variable for the rest of the non-detects with LOD => 25th percentile was included.
